# Progesterone Therapy, Endothelial Function and Cardiovascular Risk Factors: A 3-Month Randomized, Placebo-Controlled Trial in Healthy Early Postmenopausal Women

**DOI:** 10.1371/journal.pone.0084698

**Published:** 2014-01-21

**Authors:** Jerilynn C. Prior, Thomas G. Elliott, Eric Norman, Vesna Stajic, Christine L. Hitchcock

**Affiliations:** 1 Centre for Menstrual Cycle and Ovulation Research (CeMCOR), University of British Columbia, Vancouver, British Columbia, Canada; 2 Division of Endocrinology, Department of Medicine, University of British Columbia, Vancouver, British Columbia, Canada; 3 School of Population and Public Health, University of British Columbia and Vancouver Coastal Health Research Institute, Vancouver, British Columbia, Canada; University of Valencia, Spain

## Abstract

**Background:**

Progesterone is effective treatment for hot flushes/night sweats. The cardiovascular effects of progesterone therapy are unknown but evidence suggests that premenopausal normal estradiol with also normal progesterone levels may provide later cardiovascular protection. We compared the effects of progesterone to placebo on endothelial function, weight, blood pressure, metabolism, lipids, inflammation and coagulation.

**Methods and Results:**

We conducted a randomized, double-blind, 3-month placebo-controlled trial of progesterone (300 mg daily) among 133 healthy postmenopausal women in Vancouver, Canada from 2003–2009. Endothelial function by venous occlusion plethysmography was a planned primary outcome. Enrolled women were 1–11 y since last menstruation, not using hormones (for >6 months), non-smoking, without diabetes, hypertension, heart disease or their medications. Randomized (1∶1) women (55±4 years, body mass index 25±3) initially had normal blood pressure, fasting lipid, glucose and electrocardiogram results. Endothelial function (% forearm blood flow above saline) was not changed with progesterone (487±189%, n = 18) compared with placebo (408±278%, n = 16) (95% CI diff [−74 to 232], P = 0.30). Progesterone (n = 65) and placebo (n = 47) groups had similar changes in systolic and diastolic blood pressure, resting heart rate, weight, body mass index, waist circumference, total cholesterol, low-density lipoprotein cholesterol and triglyceride levels. High-density lipoprotein was lower (−0.14 mmol/L, P = 0.001) on progesterone compared with placebo. Fasting glucose, hs-C-reactive protein, albumin and D-dimer changes were all comparable to placebo. Framingham General Cardiovascular Risk Profile scores were initially low and remained low with progesterone therapy and not statistically different from placebo.

**Conclusions:**

Results indicate that progesterone has short-term cardiovascular safety. Endothelial function, weight, blood pressure, waist circumference, inflammation and coagulation were unchanged as were lipids except for HDL-C. The statistically significant decrease in HDL-C levels was not clinically important (based on lack of Cardiovascular Risk Profile change).

**Trial Registration:**

ClinicalTrials.gov NCT00152438

## Introduction

Oral micronized progesterone has recently been shown to be an effective therapy for postmenopausal hot flushes and night sweats [1]. The cardiovascular system (CVS) risks associated with menopausal ovarian hormone therapy (OHT) are of concern to clinicians and to women considering their use; many women have now discontinued taking OHT [2]–[4]. Although progestins have shown CVS risks, there is no class effect of progestogens on the CVS [5] and randomized controlled trial (RCT) evidence shows that oral micronized progesterone has less negative effects on high density lipoprotein cholesterol (HDL-C) levels than medroxyprogesterone [6]. Accordingly, we included a number of planned cardiovascular outcomes in a randomized controlled trial of progesterone for hot flush treatment. We also sought to understand whether hot flushes themselves were associated with CVS risks. Baseline CVS risk marker data from this trial showed minor relationships that appeared to vary by whether vasomotor symptoms occurred during the day or during sleep [7].

The hormones of the menstrual cycle are thought to protect women from cardiovascular risk [8]. Women's risk for CVS disease increases at an older age than does men's [9]; this is often attributed to “premenopausal hormonal protection”, which is particularly attributed to estradiol [10]. Although observational data [11] have long suggested that estrogen therapy (with or without a progestogen) is effective CVS disease prevention, large RCT data have shown lack of benefit [12] or harm [13], [14]. Progesterone, women's second ovarian steroid, may also contribute to premenopausal CVS protection. Younger, normally menstruating and ovulating women have high estradiol and cyclic progesterone levels. However, not all regular (estradiol-sufficient) cycles are ovulatory or produce normal cyclic progesterone levels [15]–[17]. Women with recurrent miscarriages have both lower-than-normal progesterone levels [18] and increased CVS risks [10]. There are suggestions that both normal progesterone and estradiol are necessary for premenopausal CVS protection, from a prospective cohort study in humans [19] and observational studies in non-human primates [20], [21]

There are no randomized, placebo-controlled trial (RCT) data on the effects in postmenopausal women of oral micronized progesterone alone (without estradiol) on changes in human endothelial function, lipids, metabolic markers, and inflammation or coagulation/fibrinolysis status. In one RCT, oral micronized progesterone significantly decreased blood pressure in older hypertensive men and women [22]. In our previous random-ordered cross-over trial of endothelial function during physiological dose *intra-arterial* estradiol, progesterone, estradiol-progesterone versus vehicle in healthy early postmenopausal women, progesterone-related increased forearm flow (FBF, +15%) was significantly greater than on vehicle and equivalent to increases on estradiol. The estradiol changes, however, did not reach significance versus vehicle [23].

Endothelial function by venous occlusion plethysmography [23] was the second primary outcome of our progesterone for hot flush trial [1]. Secondary CVS outcomes were weight, waist circumference, blood pressure, fasting lipids and glucose, high sensitivity C-reactive protein (hs C-reactive protein) and albumin (both inflammation markers) and D-dimer (a coagulation screen). Our primary purpose was to describe the physiological effects of oral progesterone administration on endothelial function and cardiovascular system markers to ensure its short-term cardiovascular safety as a single therapy in postmenopausal women. Our hypothesis was that progesterone would cause positive CVS marker effects in healthy postmenopausal women.

## Methods

The protocols for this trial and the CONSORT checklist are available as supporting information; see Checklist S1 and Protocols S1 and S2.

This study was assessed by the Clinical Research Ethics Board of the University of British Columbia that has reviewed and approved the clinical trial protocol and informed consent form (“H03-70088, Unfunded Research) - “Vasomotor Symptoms and Endothelial Function: Randomized Placebo-Controlled Trial of Oral Micronized Progesterone (Prometrium).” All participants provided written informed consent. This study was conducted according to the principles of the Helsinki Declaration.

### Design

This was a double-blind, randomized controlled trial that began recruitment in September, 2003 and completed participant follow-up in June, 2009 in the region surrounding an academic medical center in Vancouver, Canada. Endothelial function was a primary outcome. Secondary outcomes were weight and waist circumference, blood pressure and measures of lipid, fasting glucose, inflammation, coagulation changes and Framingham General Cardiovascular Risk Profile score. This trial's design involved 4-week run-in and 12-week experimental periods with testing at the end of the run-in and the last week of the experimental period. Each woman was equally likely to be randomized to oral micronized progesterone (3×100 mg at bedtime daily) or identical placebo [1].

### Participants and Procedures

Healthy community women were recruited using strategies that have previously been described [1], [24]. Eligibility criteria included: being within 1–11 y of their final menstrual flow (early postmenopausal), on no ovarian hormone therapy (estrogen, estradiol, progestin or progesterone) within the previous six months. In addition, before randomization, participants needed to be without CVS disease, non-smoking, BMI<35, without diabetes mellitus, hypertension and having a normal electrocardiogram (ECG), fasting lipids and glucose. Lipid inclusion criteria were: low density lipoprotein cholesterol (LDL-C) <4.5 mmol/L, triglycerides <4.5 mmol/L, total cholesterol to high density lipoprotein cholesterol (HDL-C) ratio <6.0 and fasting glucose <7 mmol/L. They were taking no hypertensive, lipid-lowering or other cardiovascular or diabetes therapies and were otherwise healthy.

Sample size was initially based on the hot flush primary objective and set at 125 but was increased to 165 in 2006 to allow samples for the coagulation sub-study. Sample size for the venous occlusion plethysmography was 25 participants per arm, chosen for 80% power to detect a 30% difference between progesterone and placebo [23].

### Randomization and Masking

Participants were randomized to active drug (progesterone) or identical placebo using simple one-to-one randomization (no stratification, no blocking). Pharmacists at a community pharmacy generated and administered the computer-generated allocation sequence. Treatment allocation was concealed from researchers until the data were cleaned and ready for analysis.

### Forearm Venous Occlusion Plethysmography Procedure

All assessments were made during the final baseline and therapy weeks. Forearm blood flow (FBF) was assessed in a quiet clinical laboratory maintained at 21 to 23 degrees Celsius (C). The primary outcome was percent increase in FBF over the preceding saline infusion at the maximal acetylcholine (ACh, nitric oxide-releasing, endothelium-dependent) dose. Women were asked to refrain from caffeine for 24 hours (h) prior to testing. Both of the participant's arms were supported at heart level. FBF was measured simultaneously on both arms as described [23]. Before each set of measurements, circulation to the hand was prevented by inflation of a wrist cuff to 10 mm Hg higher than her systolic blood pressure. For each measurement, a cuff placed on the upper arm was rapidly inflated to occlude venous egress (Model E10; DE Hokanson, Inc., Bellevue, WA, USA) for 10 seconds (sec) of every 20. All solutions were infused at 1.0 mL/minute (min) (Harvard Apparatus, South Natick, MA, USA) into the brachial artery of the non-dominant arm through a 27-gauge dental needle sealed to an epidural catheter with dental wax or gap-filling medical grade cyanoacrylate. Vasodilators, acetylcholine (ACh), stimulating endogenous nitric oxide release, and sodium nitroprusside (SNP), a nitric oxide donor, were infused in a standard manner [23]. The infusion sequence was: saline for 10–15 min, SNP at 1, 3 and 10 mcg/min for 6 min each at each concentration, saline for 10–15 min to allow flow to return to baseline, ACh at 3, 10 and 30 mcg/min for 4 min at each concentration. FBF was recorded during the last 3 minutes of each infusion. The mean of the last five FBF measurements at a given drug dose was analyzed and reported as %FBF increase.

### Blood Pressure and Morphological Measurements

Resting blood pressure and heart rate were automatically measured without a researcher present with an average of 3 (Omron Healthcare, Kyoto, Japan) or 5 measurements (BP-TRU Medical Devices, Coquitlam, BC, Canada). Each participant's blood pressure was measured with a single instrument. Height in meters (m) was measured at full inspiration in stocking feet, weight in kilograms (kg) was measured on a balance beam scale, body mass index (BMI) was calculated as m/kg^2^ and waist circumference in centimeters (cm) was measured at the natural waistline under clothing and during quiet breathing.

### Serum and Plasma Assays

Fasting serum was aliquotted, with some assayed at the time of collection by the Vancouver General Hospital laboratory using routine methods (Dimension Vista® analyzer) for the following: Total Cholesterol (repeatability 4% CV, reference range: 4.20–5.20 mmol/L), HDL-C (repeatability 0.6–1.3% CV, reference range 0.90–2.40 mmol/L), and triglycerides (repeatability 2–6% CV, normal values <1.70 mmol/L). LDL-C (reference range 2.20–3.40 mmol/L) was computed and its CV depends on the variability of the primary values. D-Dimer (STA – Liatest, Stago, France; repeatability 3–16% CV, reference range <500 FEU) was measured to screen for the presence of coagulation. Initially, fasting *capillary* glucose was only obtained for screening; subsequently plasma glucose samples were obtained at baseline and at the end of therapy and measured by the laboratory (repeatability 1–4% CV, reference range: 3.6–6.0 mmol/L). Additional serum aliquots were kept in short-term storage at −20 degrees C then transferred to −70 degree C until analysis at trial end (from 1–5 y). Sera were assayed for two inflammatory markers: high sensitivity C-reactive protein (repeatability of 0.06–0.36% CV; reference range <3 mg/L) and albumin (repeatability 0.1% CV, reference range 35–45 g/L). Albumin is an acute phase reactant inversely related to inflammation [25]. Those participants for whom data were not available had samples that were inadvertently not taken, had inadequate remaining sample for a particular analysis or that, over the six years of this trial, inadvertently became missing in transit between the laboratory and −70° storage. As described, later in the trial, we obtained plasma glucose values at both baseline and therapy assessments. A coagulation screen (D-dimer) was added more than half way through this trial.

### Framingham General Cardiovascular Risk Profile Score

We computed the Framingham General Cardiovascular Risk Profile Score [26] (in % risk) to assess women's 10-y risk of coronary disease, stroke, peripheral vascular disease and heart failure based on each woman's age, systolic BP, total cholesterol and HDL-C. As previously described, this risk profile is only assessing age, total cholesterol and HDL-C differences between those on progesterone or placebo since hypertension, diabetes and smoking were excluded by this trial's eligibility criteria.

### Statistical Analysis

Participant data are presented as mean plus or minus standard deviation (± SD) or as median (inter-quartile range). The effect of therapy on each outcome was assessed using analysis of covariance (ANCOVA), with appropriate baseline values and therapy assignment as covariates. The ACh and SNP %FBF results are presented without adjustment for multiple comparisons. All tests are two-sided. Statistical analyses were performed with Stata (StataCorp, College Station, Texas, USA, Version 9.2).

## Results

**Figure 1:**
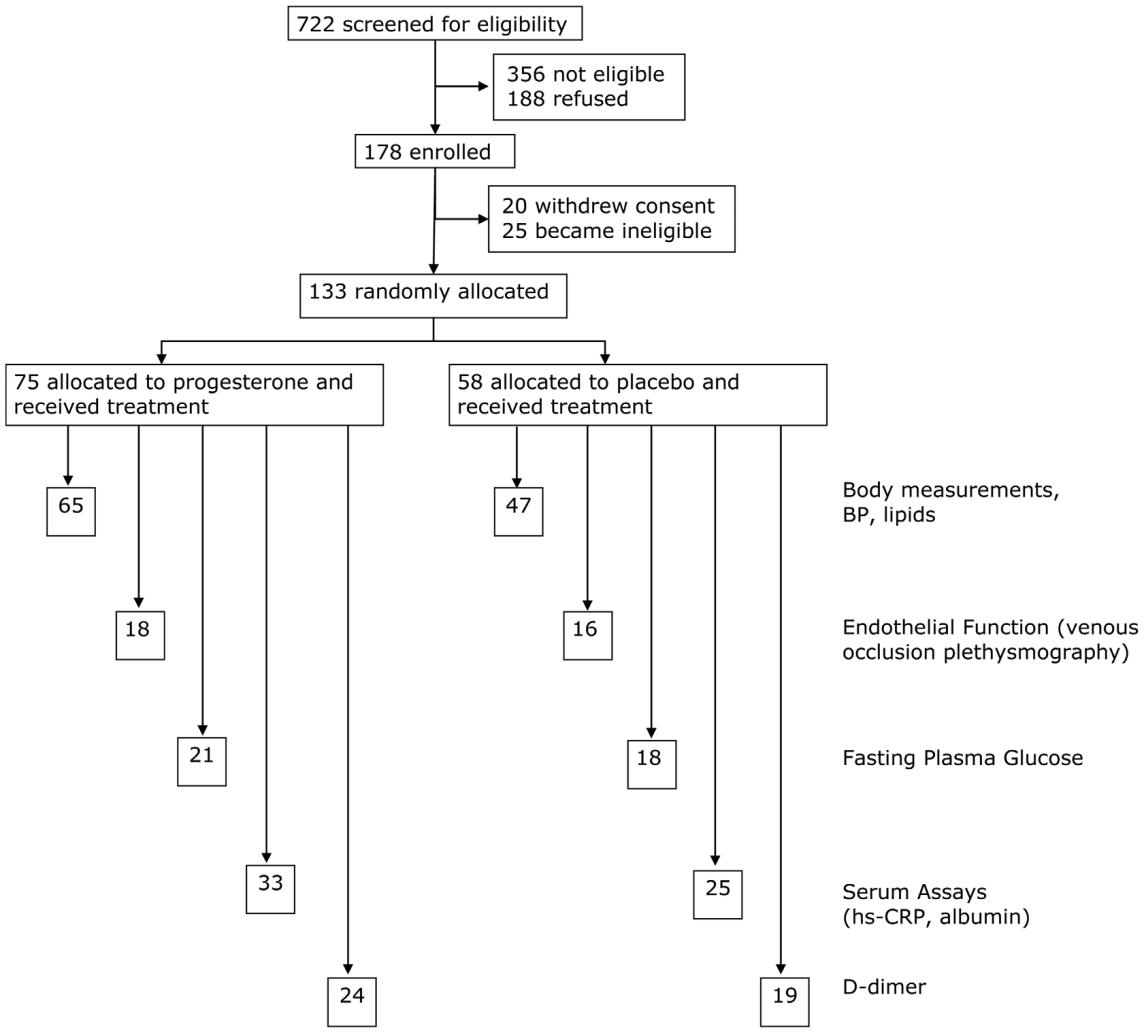
Flow Diagram Of Healthy Early Postmenopausal Participants Through The Cardiovascular Assessment Portion Of The Progesterone For Hot Flushes Randomized Controlled Trial. This shows those excluded before randomization for abnormal historical or physical examination results, those secondarily excluded because of laboratory abnormalities and those randomized to progesterone or placebo arms. Also indicated are those with analyzable venous occlusion plethysmography assessment of the endothelial function effects of progesterone or placebo, morphometric, blood pressure, lipid and inflammation data.

Cardiovascular system risk marker data were available for 112 of the 133 women in this randomized placebo-controlled hot flush trial [1] with fewer data available for endothelial function, fasting glucose, inflammation and coagulation as shown in **Figure 1**.

### Endothelial Function

Women agreeing to venous occlusion plethysmography (n = 34) were primarily white with a natural menopause [1]; those randomized to progesterone and placebo did not differ (**Table 1**). On average, women were in their mid-fifties, about three years since their last menstrual flow, of normal body size and shape and showed healthy blood pressure and heart rate values.

**Table 1:** Baseline Characteristics Of Venous Occlusion Plethysmography Participants In This Controlled Trial Of Oral Micronized Progesterone (Progesterone) Therapy For Vasomotor Symptoms.

	Progesterone (n = 18)	Placebo (n = 16)
**Age** (years)	55.8±0.9	53.9±1.0
* **y since first VMS**	4.7 (1.7, 8.3)	4.5 (2.7, 11.1)
* **y since last flow**	3.4 (2.2, 6.1)	2.4 (1.8, 4.5)
**Body Mass Index**	24.7±0.7	25.9±0.8
**Waist Circumference** (cm)	79.3±1.7	80.2±1.6
**Systolic BP** (mmHg)	118.9±3.5	116.7±3.7
**Diastolic BP** (mmHg)	67.4±2.0	69.5±2.5
**Heart rate** (beats/min)	62.9±1.7	64.8±1.6

* Median and interquartile range.

Characteristics of the 34 healthy (non-smoking, normotensive, without diabetes or heart disease) early postmenopausal women with blood pressure, lipids and ECG within clinically accepted normal ranges who were randomized to oral micronized progesterone (Progesterone, 300 mg/d at bedtime, n = 18) or Placebo (n = 16) in a hot flush trial [1] and for whom forearm blood flow was measured by standard venous occlusion plethysmography methods [23]. Data are mean ± SD unless otherwise indicated*.

**Figure 2:**
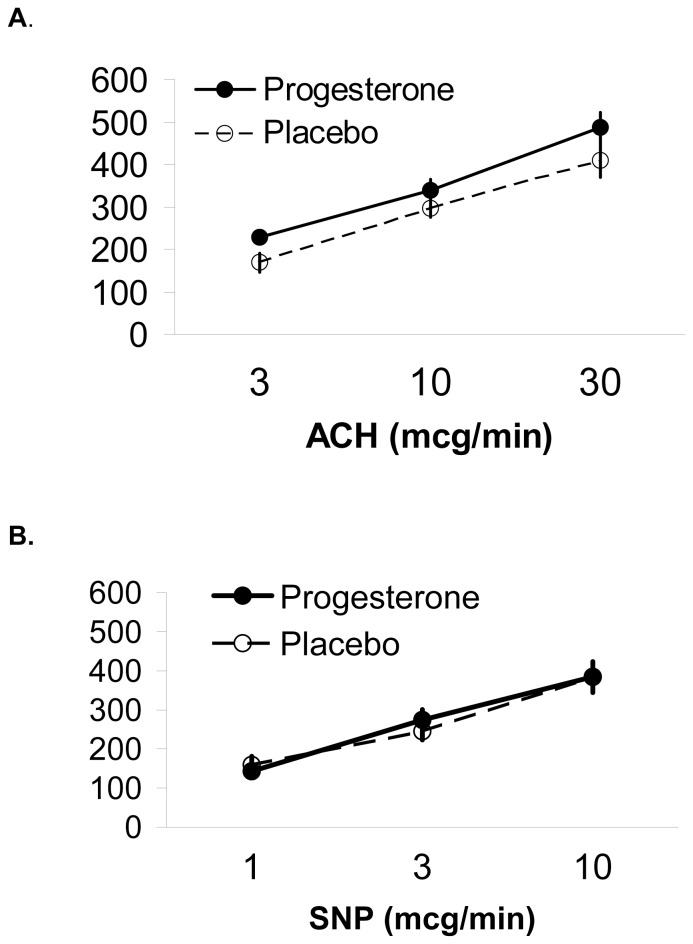
Endothelial Function on Progesterone or Placebo. Forearm venous occlusion plethysmography was performed in 34 healthy early postmenopausal women in a randomized, placebo-controlled trial of oral micronized progesterone (300 mg, Progesterone) for hot flushes. This plot shows dose responses in forearm blood flow (FBF) to intra-brachial artery infusions of vasodilators: A. nitric oxide-releasing, endothelium-dependent (acetylcholine - ACh); B. endothelium-independent (sodium nitroprusside - SNP). Data are mean ± SE of percentage (%) increase in FBF above saline baseline for those randomized to Progesterone (n = 18) and Placebo (n = 16). The 15% increase in FBF at each dose of ACh during Progesterone therapy was not statistically significant.

In both progesterone and placebo groups, FBF showed the expected dose-response to vasodilator concentration. The endothelium-dependent response to acetylcholine (ACh) was about 15% higher with progesterone than placebo, a difference (at highest concentration: 95% CI: −74, 232, p = 0.3) that was consistent across doses of ACh, but was not statistically significant (**Table 2** **,** **Figure 2**). Responses to the endothelium-independent vasodilator sodium nitroprusside (SNP) were similar on progesterone and placebo (**Table 2**).

**Table 2:** Endothelial Function Responses On Oral Micronized Progesterone (Progesterone) Or Placebo In This Randomized Controlled Trial Of Oral Micronized Progesterone (Progesterone) For Short-term Effects On The Cardiovascular System.

		Baseline			Therapy	
**Endothelium-Independent (SNP)**						
	SNP 1	SNP 3	SNP 10	SNP 1	SNP 3	SNP 10
**Progesterone**	177±23	272±36	402±59	143±14	274±26	385±37
**Placebo**	163±31	320±56	449±82	159±22	245±22	384±40
**Endothelium-Dependent (ACh)**						
	ACh 3	ACh 10	ACh 30	ACh 3	ACh 10	ACh 30
**Progesterone**	210±32	290±42	415±61	229±27	339±28	487±45
**Placebo**	201±44	312±58	406±77	169±30	298±58	408±69

Dose-response of forearm blood flow (% increase above previous saline baseline) by venous occlusion plethysmography [23] in response to increasing concentrations (in µg/min) of the endothelium-independent vasodilator, sodium nitroprusside (SNP) and the nitric oxide-releasing, endothelium-dependent vasodilator, acetylcholine (ACh). Data are mean ± standard error at baseline and following 12-weeks of randomized therapy to oral micronized progesterone (n = 18) or identical placebo (n = 16) given for hot flushes [1].

### Morphometric and Blood Pressure Measurements

These assessments were available for 112 women (progesterone, n = 65, placebo, n = 47). There were no differences in changes by therapy in weight, body mass index, waist circumference, systolic and diastolic blood pressures or in resting heart rates (**Table 3**).

**Table 3:** Cardiovascular System Marker Changes During A 12-Week Randomized Controlled Trial Of Oral Micronized Progesterone (Progesterone) For Effects On The Cardiovascular System.

	Progesterone			*Placebo*			Difference	(95% CI)	P
	baseline	therapy		baseline	therapy				
**Weight (kg)**	65.4 (8.6)	65.2 (8.8)	n = 65	66.6 (9.4)	66.3 (9)	n = 47	0.03	(−0.9 to 1.0)	0.954
**Body Mass Index (BMI)**	24.8 (2.8)	24.6 (2.9)	n = 63	25.1 (3.0)	24.7 (2.9)	n = 45	0.1	(−0.3 to 0.5)	0.541
**Waist Circumference (cm)**	78.2 (6.6)	78.3 (7.1)	n = 65	79.1 (7.0)	79.1 (7.5)	n = 47	−0.02	(−1.3 to 1.3)	0.979
**Systolic BP**	116.6 (12.2)	115.2 (12.9)	n = 64	117 (14.6)	114.4 (16.0)	n = 47	0.96	(−2.8 to 4.7)	0.615
**Diastolic BP**	71 (7.4)	70.7 (7.6)	n = 64	72.1 (7.9)	70.7 (8.5)	n = 47	0.62	(−1.7 to 3.0)	0.589
**Heart Rate**	64.2 (7.1)	67.1 (9.6)	n = 64	63.8 (6.8)	66.8 (8.1)	n = 47	−0.3	(−2.9 to 2.4)	0.833
**Fasting Glucose (mmol/L)**	4.9 (0.5)	4.9 (0.6)	n = 21	5.0 (0.6)	4.9 (0.4)	n = 18	0.1	(−0.1 to 0.3)	0.339
**Total Cholesterol (mmol/L)**	5.52 (0.86)	5.38 (0.77)	n = 63	5.43 (0.73)	5.45 (0.66)	n = 45	−0.13	(−0.30 to 0.04)	0.129
**HDL-C (mmol/L)**	1.83 (0.40)	1.70 (0.39)	n = 63	1.82 (0.42)	1.84 (0.42)	n = 45	**−0.14**	**(−0.21 to −0.07)**	**0.001**
**LDL-C (mmol/L)**	3.26 (0.70)	3.23 (0.64)	n = 63	3.15 (0.64)	3.17 (0.66)	n = 45	−0.02	(−0.18 to 0.14)	0.795
**Triglycerides (mmol/L)**	0.94 (0.54)	1.0 (0.48)	n = 63	1.0 (0.62)	0.99 (0.6)	n = 45	0.05	(−0.10 to 0.20)	0.518
**Total to HDL-C Ratio**	3.13 (0.72)	3.3 (0.77)	n = 63	3.13 (0.85)	3.12 (0.82)	n = 45	**0.18**	**(0.04 to 0.33)**	**0.013**
**C-reactive Protein (mg/L)**	1.6 (2.0)	1.4 (1.5)	n = 33	1.3 (1.6)	1.3 (1.7)	n = 25	−0.1	(−0.7 to 0.5)	0.807
**Albumin (g/L)**	41.2 (2.3)	40.8 (2.1)	n = 33	41.7 (2.0)	41.6 (2.8)	n = 25	−0.5	(−1.7 to 0.6)	0.378
**D-Dimer (µg/L FEU)**	315.7 (159.0)	352.4 (194.8)	n = 24	290.7 (86.6)	277.4 (72.8)	n = 19	53.0	(−7.3 to 113.2)	0.083
**General CVS Profile Score (%)**	4.3 (1.9)	4.3 (2.0)	n = 63	4.1 (2.1)	4.0 (2.1)	n = 44	0.1	(−0.3 to 0.6)	0.530

Cardiovascular markers are summarized as mean (SD) at baseline and at the end of 12 weeks of randomized therapy to Progesterone (300 mg at bedtime) or identical Placebo in a hot flush and night sweat treatment trial [1]. The Framingham General Cardiovascular Profile is the percent (%) risk over 10 years for acute myocardial infarction, stroke, peripheral vascular disease or heart failure (General CVS Profile Score) [26]. Difference, 95% Confidence Interval (CI) and two-tailed P report the results of analysis of covariance. Results reaching statistical significance are in **bold**.

### Fasting Serum Lipids and Plasma Glucose

**Figure 3:**
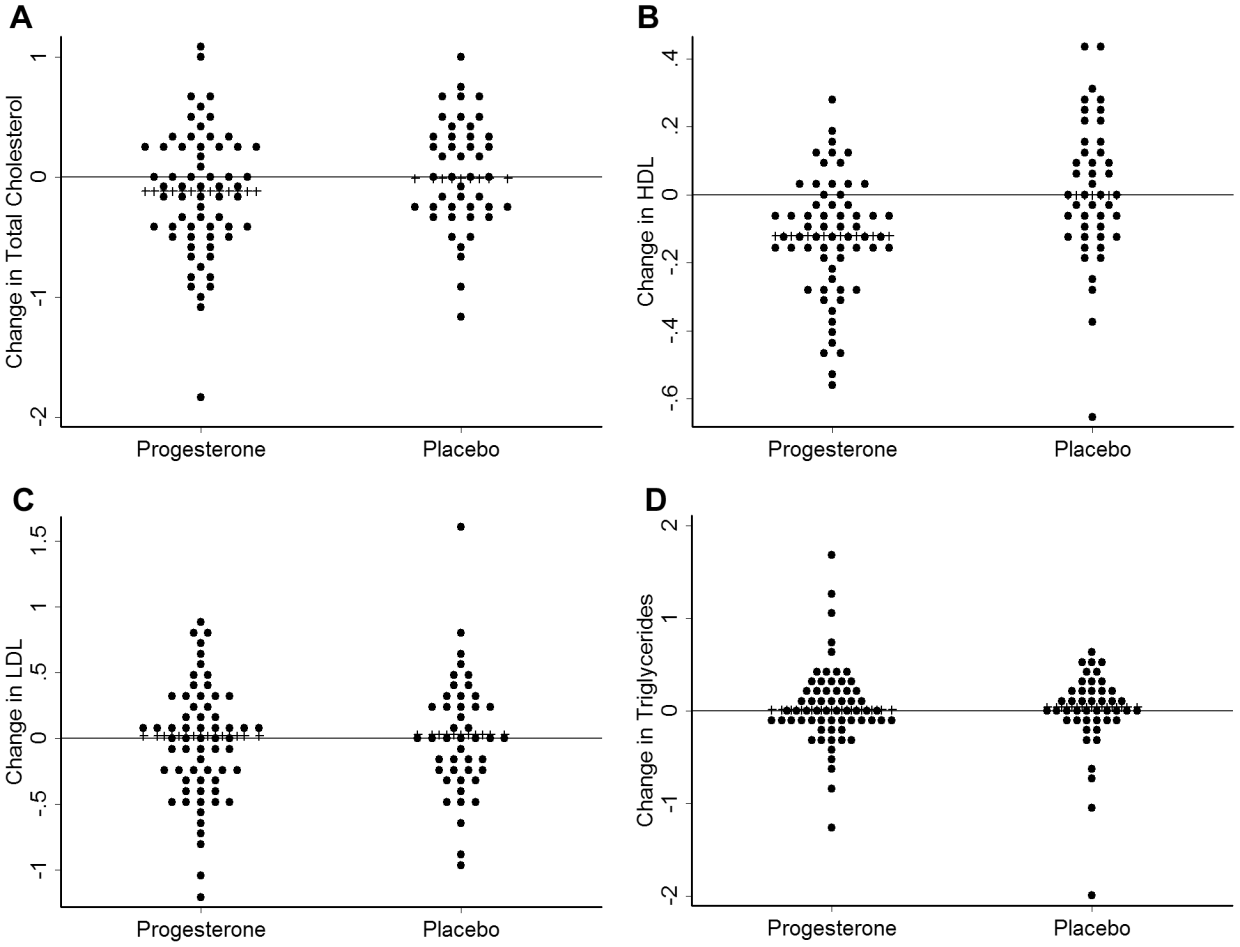
Changes in Lipids on Progesterone or Placebo. These graphs show dot plots of lipid changes in 108 healthy early postmenopausal women in a randomized controlled trial of oral micronized progesterone (Progesterone, 300 mg daily) for hot flushes between the 4-week run-in and the 12 weeks of experimental therapy. Women were randomized in a 1∶1 ratio to Progesterone (n = 63) or Placebo (n = 45). The median change is shown with a line of stars. A. Total Cholesterol changes—not different by therapy; B. High density lipoprotein cholesterol = HDC-C—the level during Progesterone therapy was significantly lower (−0.14 mmol/L, p = 0.001)( 95% CI of difference, −0.20, −0.07); C. Low density lipoprotein cholesterol = LDL-C—not different by therapy; D. Triglycerides—not different by therapy.

Lipid measurements were available for 108 women (progesterone = 63, placebo = 45) (**Figure 3**). There were no significant differences in changes in Total Cholesterol, LDL-C or triglyceride levels by experimental therapy. HDL-C values, however, decreased during progesterone therapy (difference −0.14 mmol/L, P = 0.001) (**Table 3**) (**Figure 3**). Paired plasma glucose data were available from 39 women (progesterone = 21, placebo = 18) and were not different by therapy (95% CI: −0.1 to 0.3, p = 0.34) (**Table 3**).

### Markers of Inflammation and Coagulation

Levels of C-reactive protein and albumin were available for 58 women (progesterone = 33, placebo = 25). Neither inflammation marker showed an important change; there were no differences between progesterone and placebo (**Table 3**). D-dimer was available for 43 women (progesterone = 24, placebo = 19). Results were stable within and not significantly different between progesterone and placebo (95% CI: −7.3 to 113.2 FEU) (**Table 3**).

### General Cardiovascular System Risk Profile

**Figure 4:**
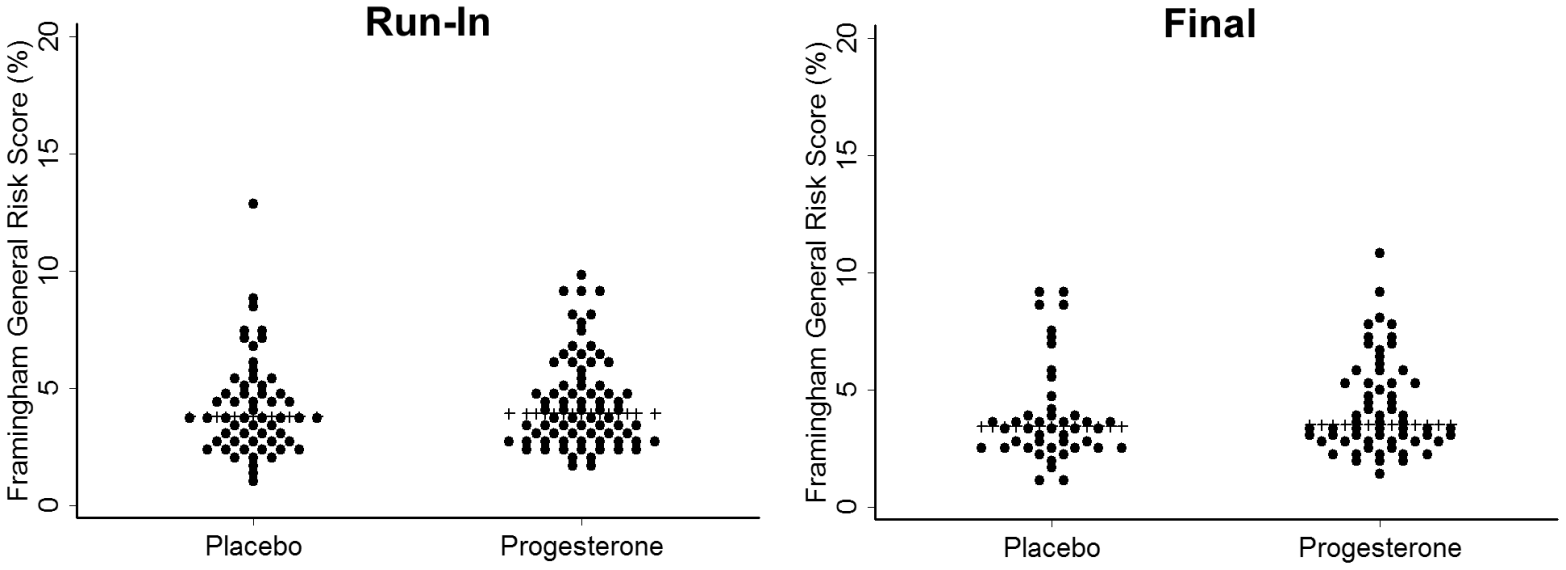
Changes in Framingham General Cardiovascular System Profile [26] 10-y Risk Score. These data are from 108 untreated healthy postmenopausal women (1–11 years since their last menstrual flow) in a randomized controlled trial of oral micronized progesterone (300 mg daily, Progesterone; n = 63) or identical placebo (Placebo, n = 45) for treatment of hot flushes/flashes and night sweats. This pair of dot-plots shows women's Framingham General CVS Profile during run-in by assignment to Progesterone or Placebo (left panel) and at the end of 12-weeks of experimental therapy (right panel). None of these differences were statistically significant.

Framingham General Cardiovascular Risk Profile [26] scores were initially low at about 4% 10-year risk in those randomized to both progesterone and placebo therapy. The difference between progesterone and placebo arms was 0.1 (95% CI: −0.3 to 0.6; p = 0.53); within-woman changes were minor and not statistically significant on progesterone (n = 63) and placebo (n = 44) (**Figure 4**).

## Discussion

These 12-wk randomized controlled trial data are the first to assess the short-term cardiovascular marker effects of oral micronized progesterone treatment in luteal phase equivalent [27] doses (300 mg/d) given alone (without estradiol or estrogen) in healthy postmenopausal women. Participants were young (in their mid-50 s), had no cardiovascular or metabolic diseases, were on no hypertension, lipid, heart or diabetes medications, were non-smoking postmenopausal women within 11 years of final menstrual flow and at a low baseline CVS risk that was similar to premenopausal women. Endothelial function was not compromised by progesterone therapy; the 15% greater endothelium-dependent increased forearm blood flow was not statistically significantly different between progesterone and placebo. These results are similar to positive and significant progesterone-related endothelial results we have previously published [23]. Progesterone did not affect body weight, abdominal obesity, systolic or diastolic blood pressure or fasting plasma glucose. There was a small but significant decrease in HDL-C levels (−7.7%), with no changes in total cholesterol, triglyceride or LDL-C levels. Progesterone did not affect inflammation markers, coagulation screen or Framingham General Cardiovascular Risk Profile compared with placebo. These data suggest that progesterone therapy tends to improve endothelial function but is largely neutral in its effects on cardiovascular markers and causes a slight decrease in HDL-C in these healthy women with low endogenous estradiol levels.

Endothelial function has previously been assessed on progesterone (as solo therapy without estrogen) only in our cross-over trial of *intra-arterial* ovarian hormones [23] and showed a similar 15% increase in flow-mediated dilatation that was statistically significantly increased compared with placebo in that study. The 15% increase in flow-mediated dilatation in this short-term trial of oral progesterone did not reach statistical significance, probably because of a lack of power. Our power calculations were based on a progesterone-placebo difference of 30%; for technical and personnel reasons we fell short of our projected sample size of 25 women per arm and only were able to test endothelial function in 34 participants in total.

We did not find the same blood pressure-lowering results as a previous study of progesterone in doses of 100–300 mg *twice* daily in hypertensive older adults [22]. This might be due to the comparatively lower blood pressure in this group of younger, normotensive women, or to the lower, bedtime-only dose of progesterone used [28], [29].

Progesterone treatment was not expected to decrease HDL-C levels. The decrease in HDL-C was positively correlated with a progesterone-related increase in Free-T4 (as previously documented [30]); this relationship only occurred in the progesterone group (results not shown). The importance of this unexpected observation is unclear. In a larger RCT, during co-therapy with oral conjugated equine estrogen, progesterone, in contrast to medroxy-progesterone acetate, did not interfere with the increased HDL-C level caused by oral estrogen [6]. However the 7.7% decrease in HDL-C levels we documented on progesterone alone may be of concern. Fasting lipids previously measured in a prospective 6-week study in which 200-mg of progesterone was given daily and bone metabolism studied in older postmenopausal women showed no changes in other lipids but a 5% decrease in HDL-C levels [31]. Although higher HDL-C levels are traditionally associated with CVS protection and lower levels with increased CVS risks, that usual relationship is not inevitable [32]. Results of a recent randomized controlled trial that raised HDL-C levels, for example, despite achieving an 18% HDL-C increase, did not decrease clinical heart disease or mortality [33]. Finally, the Framingham General CVD Risk Score [26] that incorporates historical risks (age, hypertension, diabetes, smoking) with measurements of HDL-C, total cholesterol, and systolic blood pressure did not show any changes on progesterone therapy. Thus clinical importance of this small progesterone-related decrease in HDL-C is unclear.

Progesterone caused no changes in weight, body mass index or waist circumference—no similar data to our knowledge have previously been reported. Likewise, progesterone therapy caused no changes in fasting plasma glucose or waist circumference suggesting progesterone, as opposed to some progestins, has short-term metabolic safety [5]. Progesterone exerted primarily neutral effects on fasting lipids (except for the decreased HDL-C previously discussed) versus placebo.

Increased inflammation is associated with age-related diseases including those of the cardiovascular system [34]. Statin-related lowering of C-reactive protein levels was recently shown to improve coronary artery disease outcomes in a randomized controlled trial [35]. To our knowledge there are no previous studies assessing the effects of progesterone therapy on C-reactive protein levels. However, at the end of our 1-y comparative double-blind RCT of medroxyprogesterone versus oral estrogen therapy, C-reactive protein was significantly lower on medroxyprogesterone than on conjugated estrogen [25].

Also, to our knowledge, oral micronized progesterone alone has not been assessed for effects on coagulation and fibrinolysis measures although case-control data suggest that, when combined with transdermal estrogen, it does not increase risks for venous thromboembolism [36]. In this controlled trial we were only able to measure the screening test, D-dimer, which showed wide variance, tended to increase but was not statistically different on progesterone versus placebo therapy. In this first controlled trial of the cardiovascular system marker effects of oral micronized progesterone we sought to document physiological progesterone effects in healthy postmenopausal women.

The strengths of this study are its randomized, placebo-controlled trial design, good numbers of women for a physiological study and the breadth and importance of the CVS markers we assessed. Further, this trial administered a progesterone dose (given only once a day at bedtime) that keeps the serum level at or above the luteal phase progesterone threshold for 24 hours [1], [27].

This trial also has limitations; it is a surrogate endpoint study. It measured only physiological and surrogate markers of CVS function and not cardiovascular diseases. This study might be criticized as having too short a time to show changes in weight, waist circumference, blood pressure or lipids; however, with the numbers of women we studied changes would have been visible if present. For example, it was possible to observe significant decreases in HDL-C in the 3-months on progesterone and a previous study has shown changes at two months [31]. Another major limitation is the small sample size that was available to assess endothelial function. We could also have been stricter in entry criteria by excluding all women with a body mass index >30 (obesity) or a waist circumference > 88 cm (insulin resistance). Alternatively, a less restrictive inclusion strategy would have increased our results' generalizability.

**In summary**, this RCT assessment of the endothelial function, CVS marker, metabolic, inflammatory and coagulation marker effects of oral micronized progesterone therapy has shown short-term cardiovascular system safety in healthy early postmenopausal women. There was a trend toward improved endogenous nitric-oxide dependent forearm blood flow, neutral effects on body weight, waist circumference, blood pressure, heart rate, lipids, plasma glucose, inflammation, coagulation and Framingham General Cardiovascular Risk Profile and a minor but significant decrease in HDL-C levels. In addition, in the primary hot flush trial [1], no safety issues were identified and women assigned to progesterone reported significantly improved sleep. These CVS marker results and earlier studies [23], [31] suggest that it is safe to use progesterone alone for the treatment of hot flushes and night sweats [1].

## Supporting Information

Checklist S1 The CONSORT Checklist for this randomized controlled trial is provided in this supplement.
(DOC)

Protocol S1 This is the original protocol: “Vasomotor Symptoms and Endothelial Function—a randomized placebo-controlled trial of oral micronized progesterone (Prometrium®)” August, 2004.
(DOC)

Protocol S2 This is a supplemental protocol begun when coagulation measures were added to the original Protocol S1 titled: “Appendix # 1 Oral Micronized Progesterone and Coagulation/Hemostasis: a randomized placebo-controlled trial” March, 2006.
(DOC)
