# Supplementary material for: Progesterone Therapy, Endothelial Function and Cardiovascular Risk Factors: A 3-Month Randomized, Placebo-Controlled Trial in Healthy Early Postmenopausal Women
Source: PLoS One. 2014 Jan 21;9(1):e84698. doi: 10.1371/journal.pone.0084698 (PMC3897380; doi:10.1371/journal.pone.0084698)
Supplement: Protocol S1 — This is the original protocol: “Vasomotor Symptoms and Endothelial Function—a randomized placebo-controlled trial of oral micronized progesterone (Prometrium®)” August, 2004. (DOC) [file pone.0084698.s002.doc]

***T H E U N I V E R S I T Y O F B R I T I S H C O L U M B I* A**


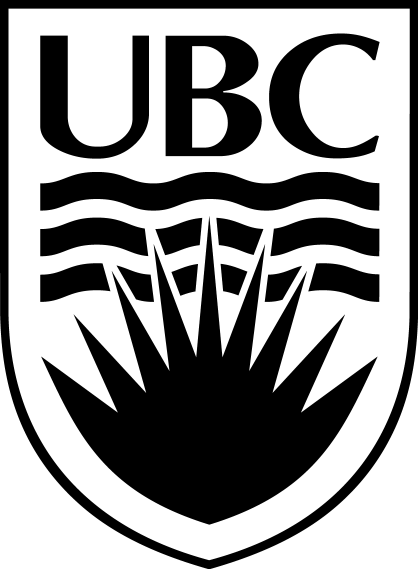


I

**UBC Department of Medicine**

*Division of Endocrinology*

*#380-575 West 8^th^ Avenue,*

*Vancouver, B.C., V5Z 1C6*

*Tel: 604- 875-5927 FAX: 604-875-5915*

jprior@vanhosp.bc.ca

## Revision Date: August 16, 2004.

**RESEARCH PROPOSAL (revised):**

# Vasomotor Symptoms and Endothelial Function—a randomized placebo-controlled trial of oral micronized progesterone (Prometrium®)

**Investigators:**

Jerilynn C. Prior BA, MD, FRCPC, Principal Investigator

## Thomas G. Elliott, MD, FRCPC, Collaborator

Eric Norman PhD, Vascular Researcher

Christine L. Hitchcock PhD, Research Associate

Yvette Vigna, BA, Research Administrator

Division of Endocrinology and Metabolism,

University of British Columbia and Vancouver Hospital,

**Financial administration:**

Funding for this study, will come from the UBC account 20R 54590 into which donations and honoraria are placed. There continues to be in kind contribution of drug and placebo for Prometrium® from Schering, Canada. This company does not control the conduct of the study.

**Introduction:**

Menopausal women often need or desire therapy to deal with night sweats or hot flashes (commonly vasomotor symptoms [VMS]). With the current evidence that estrogen with low dose progestin therapy increases the risk for pulmonary embolism, heart attacks, strokes and breast cancer (WHI 2002), women are increasingly reluctant to take it.

Progesterone and progestins are known to be effective in decreasing VMS (Albrecht, Schiff, Tulchinsky, & Ryan, 1981; Lobo, McCormick, Singer, & Roy, 1984; Paterson, 1982; Quella et al., 1998). In addition, oral micronized progesterone (Prometrium®,OMP), in a double blind randomized placebo controlled sleep study in men, increases deep sleep by 15% (Friess, Tagaya, Trachsel, Holsboer, & Rupprecht, 1997). OMP, like medroxyprogesterone (Prior, Alojado, McKay, & Vigna, 1994), is well-tolerated by menopausal women (Moyer, de Lignieres, Driguez, & Pierre, 1993). Furthermore, non-androgenic progestins do not appear to increase the risk for venous thromboembolic disease nor breast cancers, although no long safety trial has specifically tested oral micronized progesterone (OMP, Prometrium®) versus placebo. Furthermore, no placebo-controlled trial has tested OMP for VMS in menopausal women.

Women who decide to take medications for menopause and osteoporosis prophylaxis would like to know that they are also decreasing their risks for cardiovascular disease (CVD). One of the more physiological of the CVD risk indicators is abnormal function of the vascular endothelial nitric oxide system. Preliminary data in a randomized cross-over vehicle-controlled trial of intra-arterial infusion of progesterone suggests that it is similar to (or stronger than) estrogen in increasing endothelial function (Mather, Norman, Prior, & Elliott, 2000). Progesterone also decreases blood pressure (Rylance et al., 1985) and is likely to decrease weight (because of its effect to increase core temperature and metabolic rate).

**Purposes:**

The purposes of this three-month randomized double-blind placebo-controlled trial in menopausal women without risks for cardiovascular disease and with moderate VMS at baseline are:

1. To determine whether full dose daily oral micronized progesterone (OMP) increases forearm blood flow in response to infusion of acetylcholine;
2. **T**o determine, compared with a month’s pre-treatment record, whether and by how much day and night VMS will change in response to treatment with OMP or placebo;
3. **T**o determine whether OMP has any effects on change in lipids and BP compared with placebo.

**Null Hypotheses:**

1. Progesterone for three months will not increase endothelium-dependent forearm blood flow in response to acetylcholine infusion compared with the baseline before therapy or compared with placebo treatment.
2. Progesterone for three months to menopausal women with existing moderate day and night vasomotor symptoms will cause no change in VMS over time compared with placebo.
3. Hormone related quality of life (as documented by the Daily Menopause Diary©) will not differ between those randomized to Progesterone or placebo.
4. Lipid and BP changes during Progesterone will not differ significantly from those occurring on placebo.

**Design:**

Prospective four-month study with run-in observational month and three months’ randomized double blind placebo-controlled trial (RDBPCT). 125 women will be randomized to active Prometrium (300 mg) or Prometrium Placebo.

**Timeline (see flowchart in Appendix I)**—one month observational study during which they will be determined to be eligible (see below), will record **baseline experiences** on the Daily Menopause Diary ©JC Prior, 1990). After establishment of eligibility (see below), we will perform the initial forearm blood flow study (FBF, see methods) for assessment of endothelial function.

**Outcome measures:**

1. **Forearm blood flow** prospectively measured before and after three months of therapy.
2. **Vasomotor symptoms** prospectively recorded during the first month compared with changes in months one, two and three of the RDBPCT.
3. **Other hormone-related quality of life** measures on the Daily Menopause Diary©, especially self worth, sleep, and energy.
4. **Changes in two published Quality of Life instruments** the Rand SF-36 and the Menopause-specific Quality of Life (MenQoL) questionnaires
5. **Lipid, BP and weight changes**—these will provide new and important therapy effects.

**Participants:**

Menopausal women (final menstrual period one or more but less than 10 years before) without evidence of vascular disease (normal BP, without diabetes mellitus, normal cholesterol levels and non-smoker for at least a year, normal ECG) and with moderate VMS during the day and night. “Moderate VMS” are operationally defined for **daytime** as five flushes of a 1 intensity [on the 0-4 scale], 2-4 of a 2 intensity or 1-2 of a 3 intensity per week. Moderate VSM for **night** are defined as 1 flush of a 2 intensity or 2-3 of a 1 intensity per week.

Exclusion criteria:

Any menstruation in the preceding year.

A women who has had a hysterectomy without ovariectomy unless she is 60 years of age (and therefore could be confidently expected to be menopausal).

Use of ovarian hormone therapy (estrogen, progestin, progesterone or androgen) or SERM therapy (Raloxifene or Tamoxifen) in the preceding six months.

BMI over 35 or less than 20.

Mean of several pre-treatment BPs over 145/95 (supine, at rest, on no therapy).

History of abnormal cholesterol, abnormal fasting capillary glucose or a diagnosis of diabetes mellitus, any history suggestive of angina or having had an abnormal angiogram, ECG or exercise stress tests.

Finally, an abnormal baseline ECG consistent with ischemia or past infarction.

Randomization will be achieved using a random numbers table generated by computer with the code held by the dispensing pharmacy (Lancaster Medical).

All participants will be required to sign informed consent to a protocol approved by the University of British Columbia and Vancouver Hospital ethics review panels.

**Methods:**

**Forearm blood flow**—this is a classical technique called venous occlusion plethysmography (Benjamin et al., 1995) in which forearm blood flow (FBF) is assessed using changes in a strain gauge at the widest forearm points bilaterally during periods of time in which hand arterial blood flow is occluded and venous arm flow is prevented compared with baseline control infusions (Mather et al., 2000).

A brachial arterial needle is in place for 2 hours during this procedure. However, this brachial arterial needle is much smaller than those used for patients in intensive care settings. Both the PI and the plethysmography technician have undergone this procedure, and women (n=27) in a prior study (Mather et al., 2000) found it acceptable.

Impaired endothelial function is a physiological risk factor for cardiovascular disease. Acetylcholine acts in conjunction with endogenously produced nitric oxide (NO) in order to increase blood flow, so the dose-response of forearm blood flow to acetylcholine measures endothelium-dependent control of blood flow.

Women will come to the lab fasting and having had no caffeine or food for 12 hours. Their BP, fasting capillary glucose and lipid profiles will be measured and they will be sent for an ECG. Over the two hour study, standard dose-escalating concentrations of acetylcholine and sodium nitroprusside are infused intrabrachially. Acetylcholine is to assess endothelium- or nitric oxide-dependent FBF changes and sodium nitroprusside is to assess endothelium independent FBF changes. The primary outcome measure is change in mean FBF during acetylcholine infusion adjusted for changes in the opposite, un-infused arm. All plethysmography measurements will be performed twice in each woman—at baseline and after three months’ blinded intervention.

**Daily Menopause Diary©**--daily records will be obtained over four continuous months (one month of control data, and three on blinded therapy). Record-keeping using the diary will be taught using the videotape (Daily Perimenopause Diary ©1995). Videos will be made available on loan during the study. Records will be reviewed twice during the baseline month to ensure good data collection.

**Statistical analysis:**

All baseline demographic information, Forearm Blood Flow (FBF) data and Daily Menopause Diary and laboratory information will be entered into a database. Data will be cleaned and analyzed before the therapy code is broken. Residuals will be examined for non-normality and, if necessary, will be analyzed using non-parametric statistics.

Numbers calculations:

Vasomotor symptoms are the primary outcome variable for the study, quantified as a composite score of number of VMS multiplied by the intensity during both the day and night (VMS Score). This method has been used by our laboratory in the past (Van Patten et al. 2002), and has also been used extensively by Loprinzi and his colleagues (Sloan et al. 2001). In short trials in about 1000 men and women, they suggest that 50 women per arm are necessary to provide 80% power to detect a 0.58 SD difference using the same standard two-sample, two-tailed t-test that we propose to use, with an alpha level of 0.05 (Sloan et al. 2001). This corresponds to a shift of 3 units of VMS Score per day.

The total sample size for this study is 125. That is made up of 50 women per arm, plus a 25% allowance for those who discontinue or who prove to be ineligible after enrollment.

Although there is no consensus on the clinically meaningful difference for vasomotor symptoms, 3 units of VMS score is a reasonable choice. Using re-sampling techniques from existing data, (68) show that this sample size is adequate to reveal the effects that they have found with a progestin and with non-hormonal agents such as vitamin E and anti-depressants. It is expected that there will be a placebo effect of 30-45 percent—this sample size allows for that expectation.

The forearm blood flow hypothesis requires approximately 25 women per arm. To reduce the numbers, all outcomes will be compared as changes *within* women (rather than cross-sectionally by group) over time, by therapy. Conservatively, we would need to recruit 30/arm to ensure 25 women per arm completing the three-month therapy study.

The mean FBF data (which we expect will be normally distributed) will be expressed as a within-woman change from baseline. The day and night VMS data will be analyzed separately as a product of intensity and the number. Where appropriate, analyses will use parametric methods. If necessary, non-parametric tests will be used for outcome variables that are non-normal. Changes within women over time by therapy will be compared.

A two-tailed value of p <0.05 will be considered statistically significant. Statistical analysis will be performed using SAS statistical software for which our lab yearly renews the UBC site license.

**Laboratory facilities:**

Dr. Thomas G. Elliott has a well-equipped plethysmography laboratory adjacent to clinical and education areas within the Endocrinology Suite in the Echelon Centre, a facility jointly leased by Vancouver Hospital and University of British Columbia.

A conference room is equipped with a video monitor and VCR machine so that the Daily Menopause Diary video could be watched by volunteers on site with a research assistant available to answer questions.

**Research personnel:**

The division of endocrinology provides a fertile research environment. Numerous investigators, technicians and researchers interact daily. A statistical consultant, Dr. Christine Hitchcock, who is familiar with non-parametric tests and with evaluation of the Daily Menopause Diary© is available and willing to work with us on this project.

Eric Norman, PhD, is skilled in the plethysmography assessment of forearm blood flow and will be responsible for the FBF studies and their analysis. Although Dr. Norman could potentially recruit women and serve as a research assistant, he is quite committed with other trials. He will draw the fasting blood samples and prepare and batch specimens for freezing and later analysis. He will also perform capillary glucose testing.

With the assistance of Drs. Norman, Prior and Elliott, and Hitchcock, a skilled research administrator (Yvette Vigna) will supervise data entry and will perform statistical analyses. (We will hire a skilled data entry firm to perform Daily Menopause Diary entry on site—we have taught several data entry people to put Daily Menopause Diary data into the computer with speed and accuracy.)

Dr. Tom Elliott will review the FBF and clinical data, will provide independent analysis of blood flow and clinical laboratory records. He will review and revise drafts of the paper and contribute to the overall conduct of the study by assisting with recruitment, evaluating the FBF data, and advising.

Dr. Prior will supervise every aspect of this project, especially data analysis. She will be responsible for negotiations with the sponsor concerning study conduct, finances, and publication of the results.

**Significance:**

Many women discontinued estrogen therapy abruptly after the results of the Women’s Health Initiative Study (WHI, 2002) were announced in July, 2002. Most are reluctant to re-start estrogen treatment. They need effective therapy for sleep-disturbing VMS. Preliminary work from our lab has shown in a blinded parallel group trial that was not placebo-controlled that medroxyprogesterone acetate is equally effective as conjugated equine estrogen in the control of VMS. Should Progesterone be shown to significantly improve VMS over placebo (an expected 30-40% placebo response) this would be a major therapeutic advance for women’s health. Progesterone may also be expected to improve vascular function because of previous studies and it is lipid neutral in several trials. Progesterone should not be as likely to concern women because it does not increase venous thromboembolism as estrogen does, is unlikely to cause strokes as it significantly lowers BP, and does not cause abnormal vaginal bleeding or migraine headaches.

If we are able to show improved forearm blood flow after three months of Progesterone therapy this observation would suggest it has important positive vascular effects. We have previously shown that endothelium-dependent forearm blood flow is improved by acute intra-arterial progesterone infusion in physiological luteal phase doses (Mather et al., 2000).

# References:

**WHI (2002). Risks and benefits of estrogen plus progestin in healthy postmenopausal women: principal results From the Women's Health Initiative randomized controlled trial. *JAMA, 288*(3), 321-33.**

**Albrecht, B. H., Schiff, I., Tulchinsky, D., & Ryan, K. J. (1981). Objective evidence that placebo and oral medroxyprogesterone acetate therapy diminish menopausal vasomotor flushes. *American Journal Obstetrics Gynecology, 139*, 631-635.**

**Benjamin, N., Calver, A., Collier, J., Robinson, B., Vallance, P., & Webb, D. (1995). Measuring forearm blood flow and interpreting the responses to drugs and mediators. *Hypertension, 25*(5), 918-923.**

**Friess, E., Tagaya, H., Trachsel, L., Holsboer, F., & Rupprecht, R. (1997). Progesterone-induced changes in sleep in male subjects. *American Journal Physiology, 272*, E885-E891.**

**Lobo, R. A., McCormick, W., Singer, F., & Roy, S. (1984). Depo-medroxyprogesterone acetate compared with conjugated estrogens for the treatment of postmenopausal women. *Obstetrics and Gynecology, 63*, 1-5.**

**Mather, K. J., Norman, E. G., Prior, J. C., & Elliott, T. G. (2000). Preserved forearm endothelial responses with acute exposure to progesterone: a randomized cross-over trial of 17-b estradiol, progesterone, and 17-b estradiol with progesterone in healthy menopausal women. *Journal of Clinical Endocrinology and Metabolism, 85*, 4644-4649.**

**Moyer, D. L., de Lignieres, B., Driguez, P., & Pierre, J. (1993). Prevention of endometrial hyperplasia by progesterone during long-term estradiol replacement: influence of bleeding patterns and secretory changes. *Fertility and Sterility, 59*, 992-997.**

**Paterson, M. E. L. (1982). A randomized double-blind cross-over trial into the effect of norethisterone on climacteric symptoms and biochemical profiles. *British Journal Obstetrics Gynaecology, 89*, 464-472.**

**Prior, J. C., Alojado, N., McKay, D. W., & Vigna, Y. M. (1994). No adverse effects of medroxyprogesterone treatment without estrogen in postmenopausal women: double-blind, placebo-controlled, cross-over trial. *Obstetrics and Gynecology, 83*, 24-28.**

**Quella, S. K., Loprinzi, C. L., Sloan, J. F., Vaught, N. L., Dekrey, W. L., Fischer, T., Finck, G., Pierson, N., & Pisansky, T. (1998). Long term use of megestrol acetate by cancer survivors for the treatment of hot flashes. *Cancer, 82*(9), 1784-1788.**

**Rylance, P. B., Brincat, M., Lafferty, K., De Trafford, J. C., Brincat, S., Parsons, V., & Studd, J. W. (1985). Natural progesterone and antihypertensive action. *British Medical Journal, 290*, 13-14.**

**Sloan JA, Loprinzi CL, Novotny PJ, Barton DL, LaVasseur BI, Windschitl H. Methodologic lessons learned from hot flash studies. J Clin Oncol 2001; 19(23):4280-4290.**

**Van Patten CL, Olivotto IA, Chambers GK, Gelman KA, Hislop TG, Templeton E et al. Effect of soy phytoestrogens on hot flashes in postmenopausal women with breast cancer: a randomized, controlled clinical trial. J Clin Oncol 2002; 20:1449-1455.**

Initial telephone screening

1 to 10 years past LMP

no risks of heart disease

**Enrollment**

Introductory meeting (2 h)

Consent

Baseline Questionnaire

Quality of Life questionnaires:

MenQoL, SF-36

Weight, height, BP, HR, waist circumference

ECG

Video to explain the Daily Menopause Diary

**About 3 weeks after enrollment**

First Lab Visit (15 minutes)

fasting blood test (cholesterol, blood lipids)

fasting glucose

serum Progesterone level

**One month after enrollment**

First Blood Flow test (venous occlusion plethysmography) (2 hours)

plethysmography test

copy completed Daily Menopause Diaries

start taking medication (progesterone or placebo)

**About four months after enrollment**

Second Lab Visit (15 minutes)

fasting blood test (cholesterol, blood lipids)

serum Progesterone level

**About four months after enrollment**

Final Blood Flow test (venous occlusion plethysmography) (2.5 hours)

plethysmography test

copy completed Daily Menopause Diaries

return unused medication

final questionnaire

Quality of Life questionnaires:

MenQoL, SF-36

weight, BP, HR, waist circumference

Daily Menopause Diary

**Appendix I**

**Study Flowchart**
