# Supplementary material for: Progesterone Therapy, Endothelial Function and Cardiovascular Risk Factors: A 3-Month Randomized, Placebo-Controlled Trial in Healthy Early Postmenopausal Women
Source: PLoS One. 2014 Jan 21;9(1):e84698. doi: 10.1371/journal.pone.0084698 (PMC3897380; doi:10.1371/journal.pone.0084698)
Supplement: Protocol S2 — This is a supplemental protocol begun when coagulation measures were added to the original Protocol S1 titled: “Appendix # 1 Oral Micronized Progesterone and Coagulation/Hemostasis: a randomized placebo-controlled trial” March, 2006. (DOC) [file pone.0084698.s003.doc]

**Appendix # 1**

**Oral Micronized Progesterone and Coagulation/Hemostasis:**

**a randomized placebo-controlled trial**

**Jerilynn C. Prior BA, MD, FRCPC**

**Professor of Endocrinology, Director Centre for Menstrual Cycle and Ovulation Research, University of British Columbia**

**Research Question:** Does Oral Micronized Progesterone (OMP) increase markers for coagulation and/or fibrinolysis in healthy women 1-10 years from their final menstrual flow?

**Background:** There are no data about effects of oral micronized progesterone on coagulation or fibrinolysis. Although most pharmacological references caution about venous thromboembolism (VTE) with progesterone, these warnings are based on use of estrogen plus progesterone given that estrogen is known to increase VTE. The purpose of this study is to learn whether or not there are independent effects of OMP on coagulation or fibrinolysis.

Therefore, the purpose of this ancillary to an ongoing placebo-controlled three-month trial on OMP is to determine whether it has independent effects of OMP on the coagulation/fibrinolytic system.

**Hypothesis:**

Oral Micronized Progesterone in healthy menopausal women will have effects on Prothrombin Fragments 1 + 2 and other markers of coagulation/fibrinolysis that are equivalent to but no worse than the effects of placebo.

H_0_: Δ_OMP_ - Δ_placebo_ ≥ <smallest difference> detected with placebo.

**Methods:**

Design This is a sub-study added to an existing, internationally registered randomized double-blind placebo-controlled trial that has been ongoing since 2003, and which has 94 women enrolled so far, with a further 71 women still to enroll.

Study Medications: Oral micronized Progesterone as Prometrium® (supplied by Schering, Canada) and identical placebo (supplied by Besins, France) as three round 100-mg spheres at bedtime daily.

Participants:

Healthy menopausal women (1-10 years since the final menstrual flow) recruited from the general population. Exclusion and inclusion criteria are as in the main study.

Laboratory methods:

An additional 15 ml of blood will be drawn during the baseline and final blood tests.

**Coagulation markers**

Collection of the sample will be by vacutainer without using a tourniquet if possible. The first tubes will be used for serum and glucose before drawing the coagulation markers.

Three citrate tubes containing 3.8% citrate (to provide a ratio of 9:1) will be used and after centrifugation, aliquotted into 200 μl eppindorf tubes for –70-degree freezer storage before analysis. All tests will be batched so that the initial and final samples from each woman can be assayed in the same analytical run. They will be stored at -20°C for less than a week and then transferred while frozen to a -70°C freezer for storage until they are assayed.

Based on a review of previous randomized, placebo-controlled trials with coagulation/fibrinolysis markers as the outcome variables, 20 women per arm will provide sufficient power.

**Co-Investigators:**

Helena J. Teede, MD, PhD, FRACP, Professor of Womens Health at Monash University, Director of Research Jean Hailes Foundation for Women’s Health, Melbourne, Australia.

Cedric J. Carter, MB, BS, MRCP, FRCPC, Associate Professor, Pathology and Laboratory Medicine, Vancouver Hospital Centre for Blood Research, University of British Columbia, Vancouver, Canada.

Thomas J. Podor, BSc, MSc, PhD, Associate Professor, Pathology and Laboratory Medicine, St. Paul’s Hospital and University of British Columbia, Co-Director of the Dynamic Cellular Imagine and Biophysics Core of the iCAPTURE Centre for Cardiovascular and Pulmonary Research, Vancouver, Canada.

Christine L. Hitchcock, BA, MSc, PhD, Research Associate, Centre for Menstrual Cycle and Ovulation Research, University of British Columbia, Vancouver, Canada.

**Estimated timeline:**

Recruitment for this trial is ongoing and a finite number of women remain to be recruited for the VMS outcome therefore as soon as possible this protocol will be implemented. Depending on recruitment, it is anticipated that results would be ready for publication by June 2007.

**Funding:**

A donation (without restriction as to use) of $10,000 to the Centre for Menstrual Cycle and Ovulation Research will pay for the majority of the costs for these coagulation/ fibrinolysis assays. Additional support approximating $5-10,000 is still being sought. No source for funding or study materials will influence the design, analysis or results of this study.

**Publications resulting from this research:**

Dr. Helena Teede will write up the results of this study for publication in a leading general medical journal. If she writes the drafts, given her background and previous research, she will be the first author for a publication arising from this sub-study. Dr. J. C. Prior will be the senior author and Drs. Carter and Podor will be the second and third authors followed by other important CeMCOR personnel.

**Limitations:**

Given the lack of previous research on progesterone and coagulation, we have no clear guidance in planning the power calculations.

The F 1+ 2 assay in particular is very sensitive to rough venipuncture. Any value 10 SD or more above mean normal value will be eliminated from consideration. All markers will be collected only after two other tubes and if possible without a tourniquet.

Because VMS per se activate various hypothalamic neurotransmitters and glucocorticoids, they, themselves may be associated with alterations in the coagulation/fibrinolytic system. This, however, should balance out across the placebo and OMP arms because all have VMS during the control month. However, improved VMS could differentially alter responses on OMP and placebo—its direction should theoretically be toward decreased coagulation.

**Strengths:**

Randomized double-blind placebo-controlled design with three months on therapy.

Drs. Teede, Carter and Podor are well published in the field of hormone therapy and cardiovascular/coagulation function (Teede), in coagulation in general (Carter) and specifically in assay of PAI-1 (Podor). This is an important study because previous research has not studied coagulation with progesterone alone—these data will provide unique results.

(This protocol has been reviewed by the study investigators.)
